# The Use of Drones in the Area of Minimizing Health Risk during the COVID-19 Epidemic

**DOI:** 10.1007/s10846-022-01729-7

**Published:** 2022-09-30

**Authors:** Esthera Justyna Król-Całkowska, Daniel Walczak

**Affiliations:** 1 Department of International and European Law, Lazarski University, Warsaw, Poland; 2 Postgraduate Education Center, Medical University of Warsaw, Warsaw, Poland

**Keywords:** COVID-19, Fighting epidemics, Protecting public health, Aviation law, Drones in medicine

## Abstract

Despite their general availability, drones are not currently widely used in emergency medicine, distribution of medication and other medical products, as well as in epidemiological emergencies, in which limiting interpersonal contact is crucial for minimizing the public health risk. Given the current epidemiological situation, it is pertinent to consider, whether implementing activities with the use of drones can significantly contribute to minimizing health risks, and whether such initiatives are acceptable in the light of applicable legal regulations. The main objective is supported by an analysis of the usefulness of applicable provisions, indicating the direction of possible changes in existing legal regulations. Additionally, the article aims to demonstrate the feasibility of drone use in activities related to combating epidemics, as well as to emphasize their practical importance. Reports on the commercial use of drones in the distribution of goods and services have also been used as material for comprehensive analysis. Simultaneously, the article also includes data on quantities of equipment available to healthcare units in Poland for saving life and health. The present work uses the method of analysis of applicable legal regulations, as a criterion for the usefulness of existing solutions in the area of improving the quality of medical services, including preventive measures and combating the effects of an epidemic.

## 1 Introduction

Drones,[1] also known as unmanned aerial vehicles (hereinafter: UAVs), may be used not only in military technology (mainly in armed combat) but also in civilian areas, most frequently for commercial purposes - such as sports competitions or individual use for photographing events and parties. Organizations and non-military security formations use drones to ensure security when assessing humanitarian crises, overseeing natural state borders, or during reconnaissance activities in particular situations, such as traffic accidents or sudden and potentially dangerous events occurring in hard-to-reach places, e.g. forests, mountains, caves.

✉ Esthera Justyna Król-Całkowska
kancelaria.prawomed@gmail.com

Daniel Walczak
daniwalczak@gmail.com

1 Department of International and European Law, Lazarski University, Warsaw, Poland

2 Postgraduate Education Center, Medical University of Warsaw, Warsaw, Poland

In the context of the present article, the authors refer to the feasibility of using drones for healthcare purposes, which should be understood as supporting healthcare activities, as provided for in Art. 2 clause 1 item 10 of Healthcare Institutions Law [1], distribution of medicines, medical devices, dressing materials and foodstuffs for particular nutritional purposes, supporting rescue operations conducted in connection with an emergency health threat within the meaning of art. 3 pt. 8 of the Act on the State Emergency Medical Services [2] and performing activities necessary in the area of the epidemiological threat or during the epidemic.

In Polish law, the concept of epidemic threat has been defined in Art. 2 pt. 23 of the Act on preventing and combating infections and infectious diseases in humans (hereinafter referred to as UoZZ) [3], as "the legal situation introduced in a given area due to the risk of an epidemic, in order to take preventive actions specified in the Act". Furthermore, according to Art. 2 pt. 9 of UoZZ, an epidemic should be

[1] Drone - an aircraft without a pilot, controlled from the ground, used for taking photographs, dropping bombs, delivering goods, etc. – Oxford Learner's Dictionary https://www.oxfordlearnersdictionaries.com/definition/english/drone_1?q=drone(retrieved: 09.02.2020).

understood as "(...) occurrence of infections or infectious disease in a given area in a significantly greater number than in the preceding period; or occurrence of infections or infectious diseases previously not present". In the current situation related to the spread of the SARS-CoV-2 virus, using drones may be an optimal solution, e.g. in the scope of fulfilling the obligation to inform about the rules of conduct during an increased risk of infection in a given area, or to provide information on the principles of prophylaxis during the epidemic. Eliminating direct contact of medical personnel with persons exposed to infection optimizes the prophylactic measures in public health protection. In the context of combating epidemics, drones can also be used to monitor quarantine compliance, which, according to Art. 2 Pt. 12 of UoZZ should be understood as "isolation of a healthy person who had been exposed to infection, in order to prevent the spread of particularly dangerous and highly contagious diseases", as well as to ensure compliance with restrictions on movement and gatherings in public spaces. Advanced use of drones also contributes to diagnostic activities - i.e. monitoring elevated body temperature among people transiting through public areas.

The issue of drone use in healthcare has already been tackled, also by other authors [4]. In the aforementioned publication, as well as in the present article, the authors deal with the legal and purpose-related aspects of drone use in healthcare and medical emergency systems.

The vocabulary used in the present article is identical to the vocabulary contained in the legislative and regulatory provisions regulating issues related to drone use. The term "location" refers to the area of operation, and the term "flight" to flight operations. Name designations are used following the definitions contained in legal regulations, such as "first-person view operations". In the context of the present work, the authors have intentionally omitted the provisions on the procedure for examining and training entities using drones (e.g. Announcement of the President of the Civil Aviation Authority regarding training programs to obtain a qualification certificate [5], Guidelines on conducting these training programmes [6], or the Ordinance on qualification certificates [7]), considering that the narrow thematic framework of this work does not allow for discussing topics not directly related to the legitimacy of using drones for healthcare purposes.

## 2 Material and Methods

The present work analyzes the provisions of Polish law related to the possibility of using drones in the airspace for purposes of health protection, in its broadest sense. The above-mentioned provisions have been compared with the latest EU regulations, which refer to the use of drones and the conditions that must be met for their proper usage. Reports on the commercial use of drones in the area of distribution of goods and services have also been used as material for comprehensive analysis, as well as the newest data related to drone use in combating the COVID-19 pandemic.

Additionally, the article is also based on data related to quantities of equipment available to healthcare units in Poland for saving life and health. Bearing in mind the innovativeness of drone use in the area of counteracting epidemics and combating their effects, the authors have also reviewed the existing literature regarding the legitimacy of drone use in providing health services and minimizing health risk. The work is based on the method of analysis of applicable legal regulations, to gauge the usefulness of existing solutions in the area of improving the quality of medical services.

### 2.1 Legal Overview and Results

In view of the specifics of the present article, an overview of the current legal situation related to the possibility of drone use is presented below, including in the area of preventing epidemics. The overview is combined with the results, based on the analysis. The assessment of legal provisions is preceded by the presentation of the current pattern of drone use for commercial purposes, as well as the scope of their testing and the improvement of technologies related to their construction and use.

### 2.2 Patterns of Drone Use for Commercial Service Purposes

In 2014, Amazon announced its intention to use drones to deliver parcels. The oil and mining industry also uses special UAVs equipped with appropriate sensors, to map and scan terrain in 3D technology in inaccessible, cold areas (e.g. oil fields in Alaska in the Northern Slope area [8]). The Australian Swoop Aero company has conducted tests, during which vaccines for children in hard-to-reach areas of the Vanuatu Islands were delivered [9]. The fact that the commercial use of drones has found practical application in many areas [10] implies, that what serves private users or corporations can also be successfully applied for the benefit of the general community, including in the area of healthcare.

Drones are divided into those equipped with a rotor, and those carrying a fixed-wing. The first of these can take off vertically, make quick and rapid changes in direction and height, as well as hover. This category of drones is used in sports competitions, event photography, or shipment delivery (currently in its testing phase, with the permission of the relevant authorities, e.g. Amazon parcel delivery) [11]. Fixed-wing drones are much less maneuverable than rotor drones, but they usually have a larger battery capacity, which means that they can remain in the air for much longer. Some fixed-wing UAVs are smaller and can be launched from the operator's hand. Larger UAVs usually take off from a runway or a special launcher, due to their weight and large wingspan, which may reach several meters. It is worth noting that Google is testing drones aimed at extending the range of Internet access, with the use of solar power, allowing for the UAV to remain in the air for several years [12].

Drones differ in technical parameters, and above all in size. Some drones look like hummingbirds (e.g. AeroVironment's Nano Hummingbird [13]) or like a large spider with several arms (e.g. commercial drones for individual use, produced by JBL), to which rotors are attached. Some, finally, resemble miniature helicopters, or even small planes (single-wing drones). The use of drones by civil government agencies or regional authorities in some countries is often met with resistance from the local community.

For instance, Los Angeles Police Department has planned to use drones [14], which was met with opposition from citizens, who claimed that the planned activity constituted a violation of their right to privacy [15]. In 2015, Amazon [16] received the approval of the Federal Aviation Administration (FAA) for a pilot program of shipment delivery by unmanned systems. Drones in the company's fleet (according to the permit issued by the FAA) must be guided by a professional pilot, and the drone itself must remain in his sight, at a height not exceeding 122 m.

In mid-2019, Amazon announced [17], that it was introducing a new type of UAV, equipped with multiple sensors, as well as a flight control system, run by artificial intelligence (AI). Thanks to the solutions used, the drone can bypass both in the air and on the ground. This indicates that systemic drone use (both commercially and for public utility tasks) can become purposeful, focused on the needs of the individual recipient and implemented using the optimal route. It can be considered that UAVs equipped with obstacle detection and terrain recognition sensors, as well as navigation devices, can be used for medical purposes, e.g. emergency medicine delivery (e.g. in case of flood, avalanche or fire) or for a quick assessment of the situation in hard-to-reach accident sites, e.g. mountain canyons, forests, areas situated far from access roads, caves or other places objectively difficult to reach for the relevant services [18]. It should be noted that in Sweden testing of drones operating in the BVLOS system delivering AED3 defibrillators (automatic external defibrillators) are currently underway [19].

It is important to emphasize that the technology associated with the construction and use of drones is constantly improved, including, among others, by extending the battery life, improving flight range and increasing the loading capacity, while reducing the weight of the UAV itself [20].

### 2.3 Is it Possible to Use Drones in Healthcare According to Polish Law?

As researchers point out, the introduction of drones into widespread use is a new era in aviation, both civil and military [21]. In order to determine the admissibility of using drones for health protection purposes in Poland, we have analyzed Polish and EU aviation law regulations. In Poland, the main legal act regulating air traffic is the Aviation Law [22] Detailed provisions on drone use and the due permits required to fly them have been set out in the Ordinance on exemptions from certain Aviation Law provisions for certain types of aircraft, and on the conditions and requirements for the use of these devices [23] [hereinafter: RWL]. The above-mentioned Ordinance has been amended three times in relation to UAVs [24–26], which has been taken into account in the below analysis.

Polish aviation law specialists have analyzed the legal aspects of drone use, as well as the issue of drone operator liability [27, 28].

The Ordinance excludes the application of provisions that apply to drones with regard to:

a. the conditions for admission of civil aircraft to operate in the airspace,
b. obligatory entrance into the register of civil aircraft,
c. granting distinctive markings,
d. registration,
e. the obligation to have a valid airworthiness certificate,
f. certificate of fitness for aircraft parts and accessories,
g. issuing airworthiness certificates,
h. controlling compliance with environmental protection requirements regarding noise and pollution,
i. permits to perform flight operations and activities subject to certification.

The above provisions do not apply to UAVs with a total take-off weight below 150 kg (if used only for recreational or sports purposes) and drones with a take-off weight below 25 kg (if used for purposes other than leisure and sport). Therefore, the provisions regarding administrative procedures for aircraft of a strictly defined mass have been noticeably liberalized. Similar parameters also apply to the types of flights that may be carried out. For instance, VLOS (visual line of sight) flights - with a take-off weight of up to 150 kg, BVLOS (beyond visual line of sight) flights - with a take-off weight of up to 25 kg, have been exempted from the application of the Aviation Law with regard to UAV flight conditions and equipment required for manned aircraft for flight, navigation and communication. Such operations are, however, subject to the provisions set out in Annexes 6-6b to the RWL. All the listed Annexes have an identical catalog and meanings assigned to the defined terms in use.

Issues regarding drone use in healthcare have so far scarcely been analyzed in specialized literature [29–31]. When analyzing the available publications, it should be emphasized that the feasibility of UAV use in the area of broadly understood human health has been researched in the aspect of radioactive radiation distribution and contamination mapping [...]. The use of UAVs in this area was aimed at eliminating risk for personnel performing radiation mapping activities, and thus eliminating the risk in the area of public health [32]. Researchers analyzing the legitimacy of the use of drones in the area of distribution of medicinal products and medical devices argue that drones can potentially be highly reliable platforms for the delivery of medical microbiological and laboratory samples, pharmaceuticals, vaccines and medical equipment [33]. In addition, some authors believe it would be legitimate to use UAVs as medical support during mass events [34]. Many researchers have also emphasized that UAVs play a significant role in search and rescue missions, battlefield medicine, as well as in filling the gaps between third world healthcare systems and their western counterparts, as well as between major metropolitan centers and distant rural communities [35]. In the context of the actual use of drones in urgent cases, researchers emphasize that UAVs can potentially provide automated external defibrillators (AEDs) in Sweden [...]. The main discovery of the study was that receiving the AED provided by the drone was perceived as safe and less difficult than operating one's own cell phone [36].

Although the legitimacy of drone use in healthcare is not called into question, it is an innovative measure. Existing research emphasizes that "innovation refers to any good, service or idea that is perceived by someone as new" [37]. Although innovation can theoretically mean everything that is perceived by people as new, regardless of the objective novelty of a given idea or item, not every novelty deserves the name "innovation". In the authors' opinion, innovation should be understood as changes and modifications whose introduction contributes to real progress in a given field. Considering the patterns of civilization diseases and the incidence of sudden, life-threatening conditions, it should be considered that the use of new forms of support in the area of health protection is indeed an innovation leading to quality improvement in health services.

The use of drones in the area of health protection is important in view of the growing incidence of civilization diseases, which imply the occurrence of sudden life threatening events (e.g. strokes, heart attacks). It should be emphasized that, despite significant improvements in primary prevention and treatment over the past decades, strokes still cause the largest number of deaths and health complications. According to researchers, at the beginning of the twenty-first century, the incidence of strokes in Europe ranged from 95 to 290/100,000 per year, and the rates observed in young adults are steadily increasing [38]. In addition, due to population ageing, it is likely that the incidence of strokes will increase sharply in the coming years, while by 2025 around 1.5 million Europeans will suffer from it every year.

In the indicated context, it is innovative to use drones during crises and epidemics, including for quarantine monitoring. Given the current epidemiological situation related to the sharp increase in COVID-19 infections, drones can be used to provide diagnostic screening tests, medicinal products and septic materials, as well as for an information campaign regarding means of coping with the epidemic or quarantine. As mentioned earlier, the use of drones equipped with thermal imaging cameras allows for monitoring the temperature of the human body, which can eliminate the risk of spreading an infectious disease.

According to Australian media [39], researchers from the University of South Australia are working with representatives of Draganfly Inc. (USA) on a UAV called "pandemic drone", equipped not only with cameras, but also with specialized sensors to detect anomalies in coughing and human behavior, through a properly assigned and arranged computer algorithm. The task of drones would be to identify potentially infected persons, mainly in places such as airports, train or bus stations, cruise ships, offices or other human gatherings.

China, which became the first country in the world with an isolated outbreak of COVID-19 in Wuhan province, used UAVs to detect elevated body temperature already in the early days of the pandemic [40], and later [41] also for the rapid automatic transport of potentially infected samples, between the hospital and the laboratory where the pathogens were analyzed. *The above solution minimized the time needed to obtain diagnostic results and compensated for the staff shortages among medical personnel. In addition, as indicated by South China Morning Post* [42]*, drones are also used to enforce quarantine, by installing appropriate software in UAVs and equipping them with thermal sensors and high-resolution zoom lens, speakers and chemical sprayers for disinfecting large surfaces. The MicroMultiCopter company alone is to provide over 100 drones to 11 local governments* [43].

Below is an analysis of the regulations that apply to the use of drones, indicating which of them cannot be properly applied in the area of public health protection.

Annex 6 to the RWL [hereinafter: Z6] relates to unmanned *aircraft* with a takeoff mass not exceeding 150 kg, *used* in VLOS (visual line of sight) operations, and unmanned *aircraft* with a take-off mass not exceeding 2 kg, *used* in first-person-view operations [FPV]]. It should be noted that depending on the type and the technical capabilities of the UAV, the operator can direct it within his line of sight [VLOS] or beyond it [BVLOS], in which case it is subject to special provisions (Z6 chapter 4). FPV operations

- first-person view - should be classified as those in which the operator pilots an unmanned *aircraft* beyond his line of sight, determining its position in the airspace through real-time images sent to the ground by devices mounted on the UAV's deck. Z6 defines the options for using standard UAVs as recreational or sporting activities, which means that this Annex will not constitute a basis for supporting the area of health protection, including distribution of medicines, medical devices (tests) and septic materials.

It is crucial to emphasize that the UAV operator should exercise extreme caution and avoid any act or omission that could:

a) provoke a threat to safety, including a threat to air traffic safety,
b) obstruct air traffic,
c) disturb the peace or public order,
d) put anyone at risk.

The operator must fly the UAV in such a way as to avoid collision with another device and ensure that his device gives way to manned aircraft. It must be stressed that the operator is fully responsible for the decision to perform the flight, as well as its correctness. The appointment and participation of an observer in the performance do not release him from any responsibility for the safety of flight operations.

Z6 indicates that VLOS and FPV operations can take place under the following conditions*:*

a. In VLOS operations, the operator or at least one observer must maintain the UAV within his line of sight (with a naked eye), in order to determine its position in relation to the operator, ensuring a safe distance from other aircraft, obstacles, persons or animals.
b. In VLOS operations performed without an observer, loss of visual contact with the UAV is allowed when the operator controls flight parameters transmitted by the UAV's ground equipment.
c. In the case of FPV flight operations, the flight may be carried out while observing appropriate height and distance from the operator (up to 50 m above the ground and at a horizontal distance of not more than 200 m from the operator).

Safety is the main principle of using UAVs. The flight must take place in conditions which ensure a safe horizontal distance in all its phases from any manned aircraft, obstacle, person or animals in the event of a breakdown or loss of control of the device.

Pursuant to Z6 (chapter 4 point 4.1), the law defines horizontal distances depending on the specific type of object: not less than 100 m from the buildings of towns (settlement unit or other built-up area distinguished from other places by a separate name, or a different type description when it carries the same name, Cf: Art. 2 Pt. 4 of the Act of 29 August 2003 on official names of places and physiographic objects - Journal of Laws of the Republic of Poland 2019 item.1443, consolidated text), cities (Settlement unit with a predominantly compact construction pattern and non-agricultural functions, endowed with city rights or city status, granted as specified in separate regulations, Cf: Art. 2 Pt. 3 of the Act of 29 August 2003 on official names of places and physiographic objects- Journal of Laws of the Republic of Poland 2019 item.1443, consolidated text), housing estates (a housing complex constituting an integral part of a city or village, Cf: Art. 2 Pt. 9 of the Act of 29 August 2003 on official names of places and physiographic objects - Journal of Laws of the Republic of Poland 2019 item.1443, consolidated text) or from gatherings of people in the open air [in reference to an assembly of persons, as specified in Act of 24 July 2015 on Assembly Law - Journal of Laws of the Republic of Poland 2019 item.631, consolidated text); not less than 30 m from persons, vehicles, buildings not at the disposal or under the control of the operator) taking into account meteorological conditions, the structure and classification of airspace, and information on air traffic restrictions.

Depending on the different air traffic zones, drone flights may be subject to additional restrictions. For example, in the CTR-Control Zone of an airport, a UAV flight may take place under the conditions specified by the air traffic service provider, and in the ATZ-Aerodrome Traffic Zone - with the consent of the manager of the given zone and on the conditions stipulated by him. Similar restrictions apply to a Dangerous Area (DCT), Military Control Area (MCTR), or Military Aerodrome Traffic Zone (MATZ).

A UAV flight in a Restricted Area (R), such as the airspace located directly above a national park, is possible only with the consent of the manager of the given national park and on the conditions defined by him. Similarly, a flight over a Prohibited Area (P) can only take place with the consent of the manager of the area, and on the conditions specified by him. Also, in the border zone situated up to 15 km from the border, i.e. in the Air Defense Identification Zone (ADIZ), a UAV flight can take place once the air traffic service unit (ATS) responsible for the airspace, or the Airspace Management Cell at the Polish Air Navigation Services Agency have been informed. If the flight is going to take place in a construction area, it is necessary to obtain permission from its manager and to conduct it in accordance with the relevant safety rules. In the case of flights performed during sports competitions, the flight is conducted in accordance with the regulations specified by the organizer.

Horizontal distance limits do not apply to VLOS flight operations if the UAV's take-off mass is less than 0.6 kg (cf. Z6 chapter 4, point 4.2), as well as restrictions regarding CTR, ATZ, P, and ADIZ zones, if the flight is carried out at a distance greater than 1 km from the airport perimeter, 500 m from the premises protected by zone P, or at a height below 30 m or below the level of the highest obstacle, including trees or buildings, located within a radius of up to 100 m from the operator (Z6 Chapter 4 point 4.3).

If the UAV has a take-off mass below 25 kg, there is no need to obtain permission to fly, or agree on flight conditions over CTR and ATZ zones, if the flight takes place more than 6 km from the airport perimeter and at a height below 100 m above the ground (Z6 chapter 4 point 4.4).

Annex 6a to RWL [hereinafter: Z6a] applies to unmanned aircraft with a take-off mass not exceeding 150 kg, used only in VLOS operations, and unmanned aircraft with a take-off mass not exceeding 2 kg, used in FPV operations, used for purposes other than leisure or sports.

However, in the case of VLOS operations, it is difficult to find solutions that could ensure a rapid response of health services in situations threatening health and even life, in view of the restrictions regarding the permitted height and distance from the operator or buildings. Furthermore, concerning UAVs used for non-recreational purposes, Annex 6a to the RWL states that a UAV flight in FPV mode shall be performed at a height not exceeding 50 m, at a horizontal distance of not more than 200 m from the operator, and not less than 100 m from the buildings of towns, cities, settlements or gatherings of persons in the open air (Z6a chapter 4 point 1).

Considering the fact, that in VLOS operations the operator or at least one observer must maintain the *aircraft* within his line of sight (with a naked eye), (in order to *determine* its position in relation to the operator, ensuring a safe distance from other *aircraft,* obstacles, persons or animals), the use of drones referred to in Z6a in the area of healthcare is unlikely to bring about the desired effect. The matter preventing the use of drones for the above-mentioned purposes is primarily the speed required to reach the destination. Medical transport carried out by UAVs should be fast, efficient and, above all, it should allow covering long distances. The provisions of Annex 6a, like the provisions of Annex 6, exclude this possibility.

In order to consider the matter of drone use in healthcare support, emergency medical services, distribution of drugs, medical devices, dressings and self-service medical equipment (e.g. AED defibrillators), a detailed analysis of Annex 6b to RWL [hereinafter: Z6b] is required.

Z6b regulations apply to unmanned aircraft with a take-off mass below 25 kg, used in BVLOS operations outside of the airspace excluded from the zone open to general aviation, referred to in Art. 126 section 4 of Aviation Law. The provisions of the Annex also do not apply to unmanned aircraft with a take-off mass below 2 kg, used in FPV operations. This annex, like Z6 and Z6a, determines specific distances in relation to particular objects that must be observed when performing UAV flights. It should be noted that the restrictions arising from Z6b, relating to specific requirements for entities responsible for specific types of flights, need not be complied with.

The exemption from specific obligations is issued in the form of authorization by the President of the Civil Aviation Office, at the request of the concerned entity. An application may be submitted by an entity providing air services, which has operators as part of its team (Z6b chapter 7 point 7.1). The Annex itself specifies that the operational use of UAVs in BVLOS flights in healthcare-related activities is also recognized as specialized flights. The responsibility of the BVLOS operator is analogous to the responsibility of the VLOS operator, also prohibiting acts or omissions that could impede or prevent the safe operation of the flight and the observation or monitoring of the UAV environment by means of devices mounted on its deck or as part of its ground equipment (cameras, transponder), or other flight safety devices, allowing to keep a safe distance from other aircraft or obstacles.

Drone flights can be carried out both by public entities (performing tasks on behalf of the state or local government), as well as by private ones. A necessary requirement to perform a flight is the consent of the President of the Civil Aviation Authority to carry out a specific type of operation. Therefore, it should be considered whether flights aimed at supporting healthcare can be carried out in an emergency mode, which is an imminent component of effective rescue operations, or actions aimed at minimizing health risk in an epidemic. As a rule, Annex 6b to RWL stipulates that flights may be performed once the air traffic service provider has published the information on planned and implemented UAV flights.

The air traffic service provider publishes information about planned UAV flights:

a. for operational, specialized, automatic and training flights - at least 2 days before the flight date,
b. for operational flights, including those related to the healthcare system - on the day of the flight, if it was not possible to plan the flight in advance.

It should be emphasized that a planned flight must be notified 7 days before the day of its performance. In the case of operational flights, this prior notification is not required, if it was not possible to plan the flight in advance. However, an entity operating this type of flight must immediately notify the air traffic service provider of its intention to perform it.

Bearing in mind the discretion of the decision of the President of the Civil Aviation Authority regarding the exemption from certain obligations arising from Z6b, it is noteworthy that such restrictions exclude the possibility of using drones in emergencies. Drone use in crisis and unforeseen events, such as combating the effects of an epidemic, depends on the liberalization of the applicable law.

Due to the risks involved, and to ensure the safety of people on the ground, BVLOS flights and drone requirements have been regulated in detail. In addition to adequate lighting, UAVs must be equipped with devices or systems mounted on their deck, or as part of their ground equipment, which allow them to keep and continuously monitor the established flight parameters, including flight track and speed, altitude (through a barometric altimeter), the remaining charge of the supply batteries, the degree of fuel consumption, as well as the quality and strength of the communication signal between the UAV and the remote control station. The device should also facilitate the location of the UAV, also in the event of a failure, and automatically execute the emergency procedure, consisting in terminating the flight by emergency landing, continuing it along the preprogrammed route, or arriving at a designated place.

Z6b also defines the emergency procedure for BLOVS operations, i.e. in the event of loss of control over the UAV, or the loss of the UAV itself. In this case, the operator immediately notifies air traffic services by means of distance communication and he is obliged to attempt to regain contact with the drone. When reporting to the air traffic services, he must indicate the current and last location of the UAV, the moment of loss of communication, the last recorded speed, altitude and course, and the estimated time remaining until the aircraft runs out of fuel, or power in the batteries supplying the propulsion system. The researchers rightly point out that [44]: "Thanks to user-friendly regulations and a step-by-step approach, Polish airspace has become more accessible to drone operators. The operational experience which was been gathered under the national legislation has allowed for the building of a drone "airmanship", and this will be helpful in the preparation for the new EU common rules in the future.".

## 2.4 European Union Regulations as Part of the Unification of Provisions Regarding the Use of UAVs

On July 1st, 2019, the Commission Delegated Regulation (EU) 2019/945 of 12 March 2019 (Journal of Laws,UE.2019.152.1) on unmanned aircraft systems and on third-country operators of unmanned aircraft systems came into force.

The Regulation lays down general requirements for the manufacture, import, distribution and certification of UAVs, produced both in the EU and imported from third countries.

It divides drones into classes from C0 to C4, differentiating them in terms of mass and flight range (both altitude and speed), as well as technical requirements, such as GPS equipment.

The regulation also sets out requirements for the certification of unmanned aerial systems (UAVs and remote control systems), operator certification and, where applicable, UAV pilot licensing.

On July 1st, 2020, the EU Commission Implementing Regulation (2019/947) of 24 May 2019 on the rules and procedures for the operation of unmanned aircraft (Jounal of Laws, UE.L.2019.152.45) came into force into direct application.

Starting from December 31, 2020, the first step that any drone operator needs to take is to register in the country where they live or where they have their principal place of business.

New categories of air operations will also be introduced (Open, Specific and Certified), modifying the BVLOS and VLOS flight categorization currently used in many countries (including Poland). The Open category applies to VLOS operations, which do not require authorization if they meet the following conditions: the UAV's take-off mass is below 25 kg, the flight takes place at a maximum altitude of 120 m above the terrain (or above an obstacle higher than 120 m), where the risk to third parties is close to zero. This category contains additional 3 flight subcategories: A1, A2, A3. Subcategory A1 relates flights over people, which are allowed only for drones with the least risk of harming persons remaining on the ground. Therefore, such flights will only be possible for drones under 250 g and those that are officially recognized by the EU as toys [45]. The regulations on the safe use of UAVs are of particular importance [46]. UAVs weighing more than 250 g, but not exceeding 900 g, will be able to accidentally fly over a person remaining on the ground, but the operator must reduce the time of flying over a person to a minimum, as soon as he notices such a situation. In spite of meeting the weight requirements and/or being classified as toys, such UAVs may not fly over gatherings of people in the open air.

Subcategory A2 allows for the possibility of flying not closer than 30 m away horizontally from bystanders, if the horizontal speed of the flight exceeds 5 m/s or not closer than 5 m if the above speed mode is technically restricted for the given UAV. Subcategory A3 relates to the use of UAVs outside populated and residential areas, e.g. in empty fields or meadows. The "Specific" Category includes VLOS and BVLOS type operations, which require authorization from the competent authority due to posing a greater risk to persons. This category applies if all the conditions set out for the Open Category are not met. The authorization may refer to a single operation, a group of operations, standard operation scenarios or the approval of a certificate issued to an operator. The Certified Category also includes VLOS and BVLOS operations that require a UAS certification, based on the Delegated Regulation (EU) 2019/945, as well as operator certification and, where applicable, a UAV pilot license. This category covers operations that carry a high risk for bystanders - comparable to the risk of flying manned aircraft. The impact of Community regulations on the drone flight rules in the Member States has been analyzed in the existing literature [44].

It should be noted that since June 2021 Polish UAVO (unmanned aerial vehicle operator) qualifications have been transformed into EU qualifications for UAV operators (drone pilot competence certificates).

It must be noted that on April 22, 2021, the European Commission adopted the U-Space Regulation (Commission Implementing Regulation (EU) 2021/664 of 22 April 2021 on a regulatory framework for the U-space, Journal of Laws, UE. L.139) which will enter into force on January 26, 2023. This regulation shall apply to operators of unmanned aircraft systems (hereinafter: UAS), U-space service providers, providers of common information services.

When operating in the U-space airspace, USA operators shall: ensure that the UAS comply with the capabilities and performance requirements; ensure that during their operations, the necessary U-space services are used, and their requirements complied with; comply with the applicable operational conditions and airspace constraints.

The space called U-Space is the airspace in which it will be possible to use UAS. The U-space service provides logistics and IT support to UAS in space and ensures safety of people and property on the ground. UAS will need to have visual and digital identifiers that allow the U-space system to identify them. The purpose of the regulation is to harmonize activities within the community in order to strengthen this sector of the economy, that consists of the UAS unmanned aerial vehicle market.

### 2.5 The Feasibility of Drone Use for Combating the Effects of the Epidemic in Poland

According to the data of the Central Statistical Office, in 2018 there were 1541 emergency medical teams in Poland. In relation to the total number of communes (2477), for every 100,000 inhabitants, in 2018 there were 4 emergency medical teams [47]. This indicates that almost half of the communes currently lack a medical rescue team. Drones intended for rescue operations should have the necessary signaling, navigation and communication devices that will allow medical personnel to communicate with the affected persons. The law should allow for freedom in the scope of equipment for drones, which is necessary to conduct a given type of rescue operation. The above is particularly justified on the basis of Art. 41 Section 3 of the Act on State Emergency Medical Services [2] according to which both the use of ICT media and other tools enabling remote contact with the person conducting the rescue operation is lawful.

The law should explicitly allow the use of UAVs for the purposes of health protection in automatic flight technology, i.e. an operation in which an unmanned aircraft automatically takes off and lands in a designated place, flying along a programmed route, and the operator only exercises remote supervision over the operation while retaining the option of immediately taking over the remote control of the UAV, or another action in the event of a hazardous situation.

## 3 Summary

All the issues regarding the possibility of drone use in the area of health protection, technical restrictions and equipment parameters should be included in a proper ordinance of the relevant minister. Likewise, Z6b should also allow for a simplified procedure for drone use in healthcare, provided that entities wishing to conduct such activities meet certain conditions. The above would eliminate discretion when issuing permits by the President of the Civil Aviation Office for this type of activity. Similarly, the provisions [48, 49] related to excluding specific zones or areas where UAV flights are prohibited should allow medical operations, due to the unquestionable need to protect human life and health. Although the article raises a number of issues regarding the use of drones for the purpose of protecting human life and health, their use is fully justified in terms of monitoring wild animals, including for the purpose of controlling the spread of epidemics of infectious diseases, which is directly related to the elimination of health risk in the human population. Medical drones in a situation of saving human health or life should have priority and the privilege of moving in the airspace over other civil and military UAVs (just like ambulances on the roads - they are emergency vehicles in specific situations). This makes it all the more justified to authorize the use of medical drones, due to the obligations of states-parties to international health regulations [50] to monitor and prevent the consequences of international health concern.

**Code Availability** Not applicable.

**Author's Contribution** All authors contributed to the study conception and design. Material preparation, data collection and analysis were performed by Justyna Król-Całkowska and Daniel Walczak. The first draft of the manuscript was written by Daniel Walczak and all authors commented on previous versions of the manuscript. All authors read and approved the final manuscript. All authors read and approved the final manuscript.

### Declarations

The authors did not receive support from any organization for the submitted work. The authors have no relevant financial or non-financial interests to disclose. All authors certify that they have no affiliations with or involvement in any organization or entity with any financial interest or non-financial interest in the subject matter or materials discussed in this manuscript.

**Competing Interests** The authors have no competing interests to declare that are relevant to the content of this article.

**Ethics Approval** Not applicable.

**Consent to Participate** Not applicable.

**Consent to Publish** Not applicable.

**Open Access** This article is licensed under a Creative Commons Attribution 4.0 International License, which permits use, sharing, adaptation, distribution and reproduction in any medium or format, as long as you give appropriate credit to the original author(s) and the source, provide a link to the Creative Commons licence, and indicate if changes were made. The images or other third party material in this article are included in the article's Creative Commons licence, unless indicated otherwise in a credit line to the material. If material is not included in the article's Creative Commons licence and your intended use is not permitted by statutory regulation or exceeds the permitted use, you will need to obtain permission directly from the copyright holder. To view a copy of this licence, visit http://creativecommons.org/licenses/by/4.0/.

## Abbreviations

**Dz.U.**: Journal of Laws of the Republic of Poland [Dziennik Ustaw]
**Dz.Urz.ULC.**: The Official Journal of the Civil Aviation Authority [Dziennik Urzędowy Urzędu Lotnictwa cywilnego]
